# ESCAIDE 2022: call for abstracts

**DOI:** 10.2807/1560-7917.ES.2022.27.15.2204141

**Published:** 2022-04-14

**Authors:** Eurosurveillance editorial team

**Affiliations:** 1 European Centre for Disease Prevention and Control (ECDC), Stockholm, Sweden

The European Scientific Conference on Applied Infectious Disease Epidemiology (ESCAIDE) 2022 will be organised as a hybrid event, in Stockholm and online, from 23 to 25 November 2022. ESCAIDE 2022 welcomes abstracts in all areas applied to infectious disease and public health. More information and guidelines are available here. 

**ESCAIDE 2022 call for abstracts is open from 8 April to 18 May.**

The ESCAIDE 2022 programme is shaping up and all relevant updates are announced on the ESCAIDE website and on social media channels Facebook and Twitter @ESCAIDE. To receive regular updates on the conference by mail, please send a request to escaide.conference@ecdc.europa.eu.

